# Surface carboxylation of iron oxide nanoparticles brings reduced macrophage inflammatory response through inhibiting macrophage autophagy

**DOI:** 10.1093/rb/rbac018

**Published:** 2022-04-20

**Authors:** Di Deng, Shengxiang Fu, Zhongyuan Cai, Xiaomin Fu, Rongrong Jin, Hua Ai

**Affiliations:** National Engineering Research Center for Biomaterials, Sichuan University, No. 29 Wangjiang Road, Chengdu 610064, China; National Engineering Research Center for Biomaterials, Sichuan University, No. 29 Wangjiang Road, Chengdu 610064, China; National Engineering Research Center for Biomaterials, Sichuan University, No. 29 Wangjiang Road, Chengdu 610064, China; National Engineering Research Center for Biomaterials, Sichuan University, No. 29 Wangjiang Road, Chengdu 610064, China; National Engineering Research Center for Biomaterials, Sichuan University, No. 29 Wangjiang Road, Chengdu 610064, China; National Engineering Research Center for Biomaterials, Sichuan University, No. 29 Wangjiang Road, Chengdu 610064, China; Department of Radiology, West China Hospital, Sichuan University, Chengdu 610041, China

**Keywords:** autophagy, inflammation, superparamagnetic iron oxide, carboxylation

## Abstract

Macrophage autophagy is a common biological response triggered by nanomaterials, which is closely related to the regulation of inflammation. Superparamagnetic iron oxide (SPIO) nanoparticles have been used for study of autophagy response due to their broad biomedical applications. However, few reports have focused on how to regulate the macrophage autophagy response induced by SPIO nanoparticles. In this study, SPIO nanoparticles grafted with carboxyl groups were synthesized and for the comparison of macrophage autophagy with unmodified nanoparticles. The study on the correlation between autophagy and inflammation induced by the two kinds of SPIO nanoparticles was also included, and the one that grafted with carboxyl groups shows a reduction of autophagy and thereby caused a milder inflammatory response. We proposed that the increased amount of albumin adsorption on the surface of carboxylated SPIO nanoparticles, a protein previously proven to attenuate autophagy, can be considered an important reason for reducing autophagy and inflammation. In general, the carboxyl modification of SPIO nanoparticles has been demonstrated to reduce inflammation by inhibiting macrophage autophagy, which may provide some insights for the design of nanomaterials in the future.

## Graphical Abstract

**Figure:**
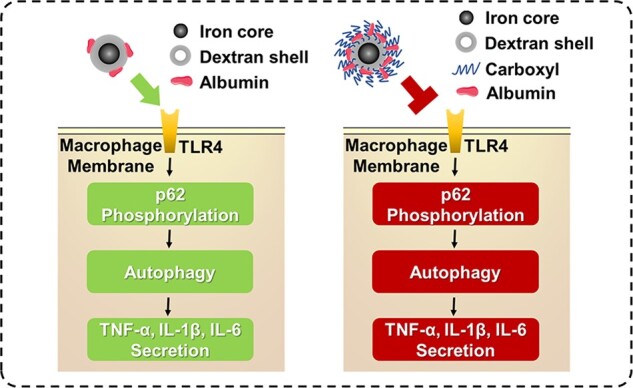

## Introduction

Nanotechnology has brought great advances in the development of biomedical engineering, providing more opportunities for diagnosis and therapy options. The construction of nanomaterials as drug carriers and imaging agents has gained great enthusiasm in the field of biomedical engineering [1]. Intravenous injection of nanoparticles is a common administration route and the particles are mainly recognized and retained by macrophages, which may induce immune responses resulting in safety risks. Therefore, in-depth study of the interaction between macrophages and nanoparticles is of vital importance to assess the biocompatibility and biosafety of nanoparticles, thereby providing guidance for improving the performance of nanomaterials [2].

Recent studies have shown that autophagy is a ubiquitous response of cells to nanomaterials, and macrophage autophagy has become a favorable entry point for studying the bioeffects between nanoparticles and the body due to its association with various physiological or pathological reactions, such as the removal of invading organisms, the occurrence and development of inflammation, the growth of atherosclerotic plaques, tumor-related macrophage polarization phenotype regulation, etc. [3–7]. Studies also demonstrated that the autophagy induced by nanoparticles can be modified by particle size, composition, surface charge, surface chemical groups and other physical and chemical properties of the material, so as to regulate cellular behaviors [8]. Although the mechanism and function of nanomaterial-induced autophagy of macrophages are still unclear, it may be used to improve the biosafety of the materials by regulating the autophagy of macrophages.

Superparamagnetic iron oxide (SPIO) nanoparticles, a widely studied inorganic nanomaterial, have broad application in biomedical engineering, such as MRI imaging, drug delivery, cell tracking and gene therapy [9]. Therefore, extensive demonstrations of SPIO nanoparticles’ biosafety have been carried out from the aspects of cells, signal molecules and genes [10, 11]. However, the evaluation of autophagy in macrophages as well as the methods to improve biosafety by regulating macrophage autophagy is rarely studied, since there has been a lot of research SPIO-induced autophagy in other types of cells [12–15]. In this study, SPIO and carboxylated SPIO (SPIO-C) with two particle sizes of 20 and 50 nm were prepared for the autophagy evaluation. We have studied the difference in macrophage autophagy induced by SPIO and SPIO-C, and investigated the relationship between macrophage autophagy and inflammation, thereby proposed a surface modification method to improve the biosafety of SPIO by grafting carboxyl groups. Macrophage autophagy can be considered as an indispensable supplement to the safety evaluation of biomaterials, providing more insights for development of better nano-formulations.

## Materials and methods

### Synthesis of SPIO nanoparticles

Dextran-10 (Sangon Biotech) and FeCl_3_·6H_2_O (Aladdin) were dissolved in 20 ml of UP water, and then 0.282 g of FeCl_2_·4H_2_O (Aladdin) was quickly dissolved in the above solution in an argon atmosphere with the oxygen in the system discharged. Under the condition of ice bath and 1000 rpm mechanical stirring, 2 ml ammonia (25–28%, Chron Chemicals) was dropwise added to the solution. After stirring for 5 min, the ice bath was removed and oil bath was introduced. As the temperature of the reaction system reached 80°C, the reaction continued for 90 min. After that, the system was cooled to room temperature and then centrifuged at 1500 rcf for 15 min. Finally, the supernatant was centrifuged again at 5000 rcf for 30 min and dialyzed with UP water for 3 days with a 100 000 kDa dialysis bag. The feed ratio for carboxylation of SPIO nanoparticles with different particle size was shown in Table 1. Dextran-SPIO nanoparticles with the particle size of 20 nm was named as SPIO(20), and those with the particle size of 50 nm was named as SPIO(50).

**Table 1.** Different feed ratio for synthesis of SPIO nanoparticles with different particle size

	Dextran-10 (g)	FeCl_3_·6H_2_O (g)	FeCl_2_·4H_2_O (g)
SPIO(20)	10.015	0.697	0.282
SPIO(50)	5.19	1.589	0.668

The SPIO nanoparticles with carboxyl grafting were prepared subsequently. Briefly, the prepared nanoparticles were reacted with different feed ratios of bromoacetic acid (Aladdin) and sodium hydroxide (Aladdin) at 50°C for 24 h, then the product was collected and placed in a 10 000 kDa dialysis bag and dialyzed with UP water for 3 days. The feed ratio for carboxylation of SPIO nanoparticles with different particle size was shown in Table 2, and the SPIO-C nanoparticles with different sizes were named as SPIO(20)-C and SPIO(50)-C.

**Table 2.** Different feed ratio for carboxylation of SPIO nanoparticles with different particle size

	Fe content of SPIO nanoparticles (μg/l)	Bromoacetic acid (M)	NaOH (M)
SPIO(20)-C	4.5	0.2	2
SPIO(50)-C	8.6	0.55	2

### Characterization of modified SPIO nanoparticles

The morphology of SPIO nanoparticles was observed by a TEM (H-600, Hitachi). The size distribution and ζ-potential of SPIO nanoparticles was measured by a Zetasizer (Nano-ZS90 apparatus, Malvern). The chemical groups of SPO nanoparticles were verified by FTIR (Nicolet 6700, Thermo Fisher Scientific).

### *In vitro* cell experiment

Raw 264.7 was applied in all *in vitro* cell experiments. Cells were cultured in RPMI 1640 medium containing 10% FBS by volume, 100 U/ml of penicillin and 100 μg/ml of streptomycin and placed in a cell incubator with a constant temperature of 37°C and a constant carbon dioxide concentration of 5%. The Raw 264.7 stably expressing GFP-LC3 was observed by a CLSM (LSM780, Zeiss) at an excitation wavelength of 488 nm after incubated with SPIO nanoparticles for 24 h to characterize the process of autophagy. The cells for western blotting and RT-qPCR were collected by using trypsin after co-cultured with SPIO nanoparticles for 24 h.

### Western blot analysis

The protein was extracted by lysing the cells with 1× RIPA buffer containing protease inhibitor and phosphatase inhibitor, and the protein content of the supernatant was quantified by a BCA Protein Assay Kit (Beyotime). Then, the supernatant with loading buffer added after boiled for 3 min was loaded to the polyacrylamide gel for SDS-PAGE. After that, the gel was taken out and soaked in the transfer buffer for 5 min to balance the ionic strength. The gel was then covered by a polyvinylidene fluoride membrane and a filter paper and tightened between the cathode plate and the anode plate for transferring the protein. After the protein was transferred to the PDVF membrane, the membrane was blocked with TBST buffer adding 5% non-fat milk by volume and then incubated with primary antibodies against LC3 (Cell Signaling Technology), p62 (Abcam), TNF-α (Abcam), IL-1β (Abcam), IL-6 (Abcam) or β-actin (Santa Cruz Biotechnology) and subsequently with secondary antibodies conjugated with Alexa dyes in order. Finally, the protein band on the membrane was visualized with a BeyoECL Moon (Beyotime) and a Western Blot Imaging System (ChemiDoc Touch, Bio-Rad).

### RNA RT-qPCR

The RNA in cells or tissue was extracted by a Total RNA Isolation Kit (BioTeke), and the cDNA was prepared by 2 μg of total RNA and a Super OneStep RT-PCR Kit (BioTeke). Then, the real-time PCR was conducted by using a KAPA SYBR Fast Universal qPCR Kit (Kapa Biosystems) and tested by a Touch Real-Time PCR Detection System (CFX96, Bio-rad). During the amplification process, the fluorescent signal was collected and performed as a melting curve. The Ct value, defined as the number of PCR cycles when the fluorescence value reaches the threshold, is inversely proportional to the logarithm of the initial number of the template, which can be used as the basis for quantitation. Subsequently, according to the comparative Ct method (2-ΔΔ CT), the level of target mRNA was measured by taking the level of β-actin as an endogenous reference. The primer sequences designed for TNF-α, IL-1β, IL-6, p62 and β-actin are listed in Table 3.

**Table 3.** Primer sequences for target amplification

Amplification target	Forward	Reverse
TNF-α	CGATGGGTTGTACCTTGTCTAC	GCAGAGAGGAGGTIGACTTTC
IL-1β	GGTGTGTGACGTTCCCATTA	ATTGAGGTGGAGAGCTTTCAG
IL-6	GATAAGCTGGAGTCACAGAAGG	TTGCCGAGTAGATCTCAAAGTG
p62	GTGGTGGGAACTCGCTATAAG	GAAAGATGAGCTTGCTGTGTTC
β-actin	CTCCCTGGAGAAGAGCTATGA	CCAAGAAGGAAGGCTGGAAA

### *In vivo* experiments

All animal procedures were conducted in accordance with the Biomedical Research Ethics Committee of West China Hospital of Sichuan University. Male Balb/c mice (Dashuo, Chengdu) were used for *in vivo* experiment. The SPIO(50) and SPIO(50)-C were intravenously injected into the mice at a concentration of 25 mg Fe/kg, and the control group was treated with saline. After the SPIO nanoparticles circulated in the body for 24 h, the blood was collected for the evaluation of ALT, AST, CRE and BUN by an automatic biochemical analyzer (Chemray 800, Leidu). The liver, kidney, heart, lung and spleen were collected after the nanoparticles circulated for 24 h *in vivo*, and then fixed, embedded and sectioned for histopathology and immunohistochemistry evaluation. The tissue was extracted for western blot and RT-qPCR analysis.

### Statistical analysis

All the experiments were repeated at least three times, and the data were expressed as the mean ± standard deviation (SD). The statistical differences between groups were determined by One-way ANOVA. *P*-values was used to determine the significance of the differences between groups, and the differences were considered statistically significant once *P* < 0.05.

## Results and discussion

### Characterization of SPIO and SPIO-COOH nanoparticles

**Figure 1.**
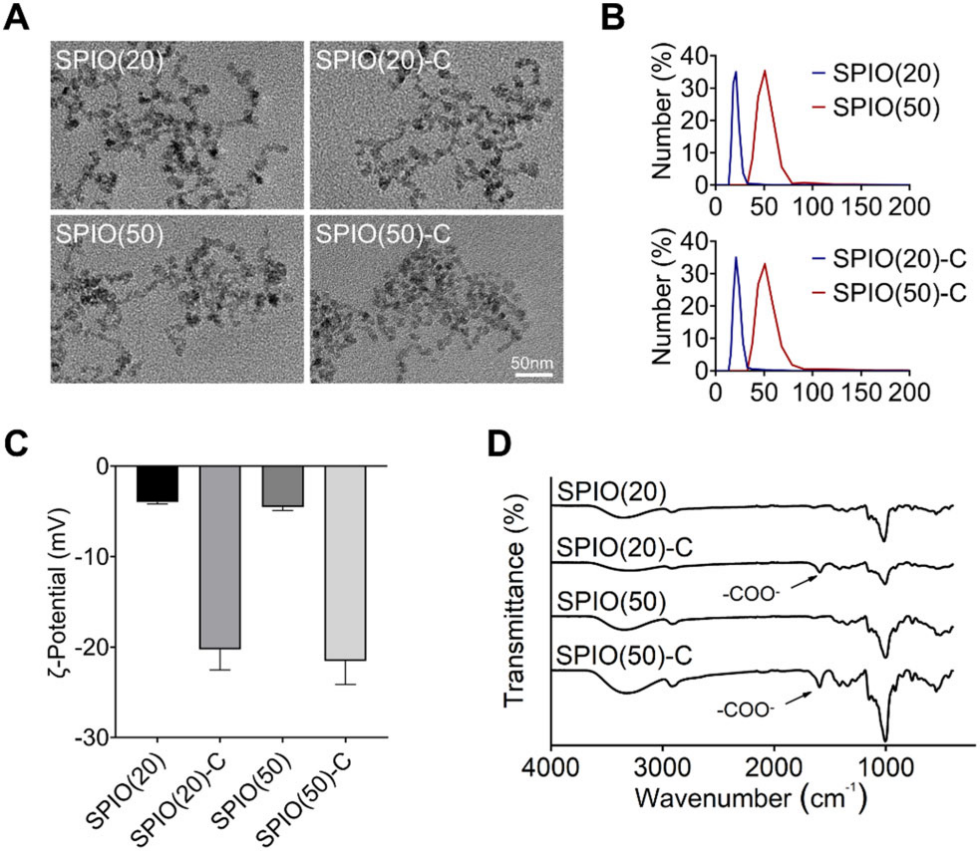
Material characterization of synthesized SPIO nanoparticles. (**A**) Morphology of synthesized SPIO nanoparticles photographed by TEM. (**B**) Hydrated size distribution of synthesized SPIO nanoparticles. (**C**) ζ-potential of synthesized SPIO nanoparticles. (**D**) FTIR spectrum of synthesized SPIO nanoparticles

Dextran-SPIO nanoparticles with average diameters of 20 and 50 nm were synthesized by the aqueous-phase co-precipitation method and named as SPIO(20) and SPIO(50). Bromoacetic acid was subsequently used to introduce carboxyl groups on the particle surface, and the resulted carboxylated nanoparticles of different sizes were named as SPIO(20)-C and SPIO(50)-C, respectively. The SPIO nanoparticles were first characterized by transmission electron microscope (TEM) to observe the morphology (Fig. 1A). The sizes of the nanoparticles were detected by dynamic light scattering (Fig. 1B) and remained stable in water (Supplementary Fig. S1A). The surface charges were determined by measuring the ζ-potential, which showed the more negative charges on the SPIO nanoparticles grafted with carboxyl **(**Fig. 1C). Then, the chemical composition of the SPIO nanoparticles was analyzed by the Fourier transform infrared spectroscopy (FTIR). The band at 1593 cm^−1^ on the FTIR spectrum corresponded to the COO^−^ stretching vibration, which further revealed the successful preparation of SPIO-C nanoparticles (Fig. 1D).

### Macrophage autophagy induced by SPIO nanoparticles

**Figure 2.**
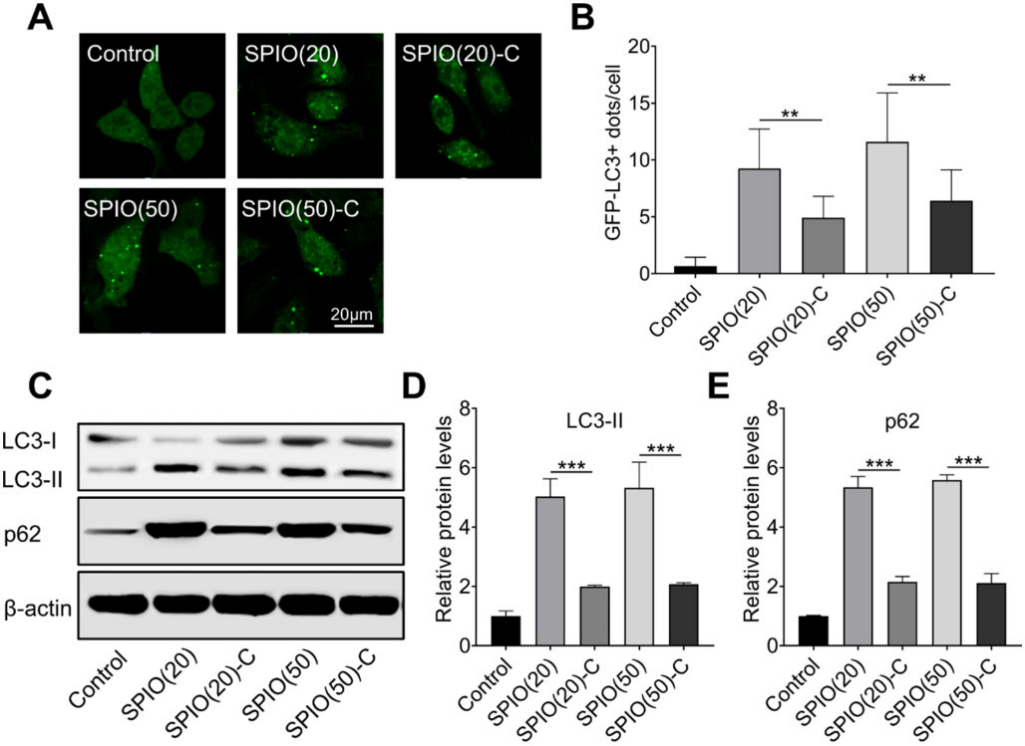
Characterization of SPIO nanoparticle induced macrophage autophagy. (**A**) CLSM image of fluorescent autophagosome in Raw264.7 with stably expression of GFP-LC3 after treated with SPIO nanoparticles for 24 h. (**B**) Statistical analysis of GFP-LC3+ puncta in Raw264.7 cells. (**C**) Western blotting of LC3 and p62 in Raw264.7 treated with SPIO nanoparticles for 24 h, β-actin was detected as a loading control. Densitometry analysis for quantifying the levels of LC3-II (**D**) and p62 (**E**) values are expressed as the mean, *n* ≥ 3. Bars show SD. **P < 0.01; ***P < 0.001

Macrophage autophagy induced by SPIO nanoparticles can be evaluated by observing fluorescent puncta in GFP-LC3 stable expression cell lines. During cell autophagy, the GFP-LC3 in the cytoplasm tends to translocate to the autophagosome membrane and form fluorescent puncta that can be observed for autophagy evaluation [16]. We initially observed the SPIO nanoparticle treated Raw 264.7 with stable expression of GFP-LC3 protein through the confocal laser scanning microscope (CLSM), and found lower amounts of LC3 puncta in SPIO-C nanoparticles-treated macrophages, which indicated the reduction of SPIO-induced autophagy in macrophages caused by grafting carboxyl groups on the nanoparticles (Fig. 2A and B). Once autophagy occurs, LC3-II and p62 will accumulate in the cytoplasm, which are generally considered as a standard for autophagy assessment [17]. Therefore, western blotting of LC3 and p62 was conducted (Fig. 2C) and quantified by densitometry analysis (Fig. 2D and E). All SPIO nanoparticle samples could cause autophagy in the macrophages, but ∼60% decrease in the expression level of LC3-II and p62 was observed in the groups treated by SPIO-C nanoparticles compared with the groups treated by unmodified SPIO nanoparticles, indicating the inhibition of SPIO-induced autophagy by grafting carboxyl groups on nanoparticle surface.

**Figure 3.**
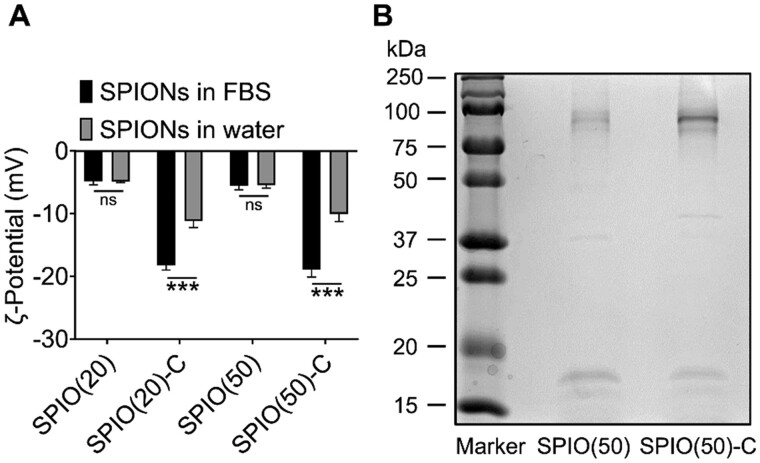
Serum protein absorption on the nanoparticle surface. (**A**) ζ-Potential changes of nanoparticles after 24 h incubation with FBS. (**B**) Electrophoresis gel of SDS-PAGE experiment on the SPIO absorbed with serum proteins. Values are expressed as the mean, *n* = 6. Bars show SD. ***P < 0.001; ns, not significant

Studies have shown that the adsorbed serum proteins on nanoparticles significantly affect the bioeffects of the materials *in vivo* [18–20]. In order to investigate whether the different levels of autophagy induced by nanoparticles with different surface properties are caused by the diverse proteins adsorption on distinct SPIO nanoparticles, the ζ-potential of SPIO nanoparticles were measured before and after incubation with the medium containing 10% fetal bovine serum (FBS) by volume. The surface charge of SPIO(20)-C and SPIO(50)-C decreased by 39% and 47%, respectively (Fig. 3A). In addition, the particle size of the SPIO nanoparticles increased by 5–10 nm after adsorbing serum protein (Supplementary Fig. S1B). SPIO(50) and SPION(50)-C that fully adsorbed serum proteins were collected by centrifugation and then subjected to sodium dodecyl sulfate–polyacrylamide gel electrophoresis (SDS-PAGE). The electrophoresis gel showed that the amount of protein adsorption on the surface of SPIO nanoparticles modified by carboxyl groups was more than that of unmodified ones (Fig. 3B), which could attribute to higher charge density and hydrophilicity of carboxylated nanoparticles [21]. The liquid chromatography tandem mass spectrometry was conducted for identifying the type of adsorbed proteins, showing that a variety of serum proteins were adsorbed on the surface of SPIO nanoparticles, including albumin, complement components, coagulation-related proteins, apolipoproteins, structural proteins, etc (Supplementary Tables S1 and S2). Previous studies have reported that albumin has the effect of inhibiting autophagy [22, 23], hence the higher amount adsorption of albumin on SPIO(50)-C than on SPIO(50) is the most likely cause of lower macrophage autophagy induced by SPIO-C nanoparticles.

**Figure 4.**
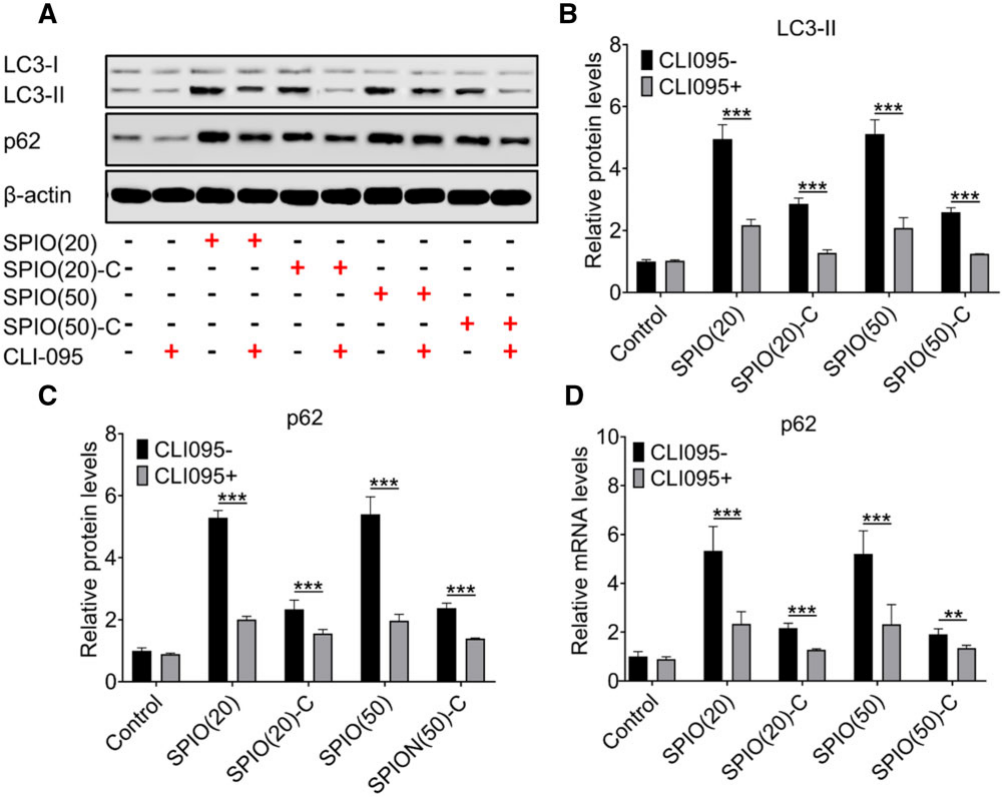
Mechanism of SPIO-induced macrophage autophagy. (**A**) Western blotting of LC3-II and p62 in Raw264.7 treated with SPIO nanoparticles for 24 h with or without adding CLI-095, β-actin was detected as a loading control. Densitometry analysis for quantifying the levels of LC3-II (**B**) and p62 (**C**). (**D**) Expression of p62 mRNA in Raw264.7 treated with SPIO nanoparticles for 24 h detected by RT-qPCR. Values are expressed as the mean, *n* = 3. Bars show SD. **P < 0.01; ***P < 0.001

To clarify the mechanism of the macrophage autophagy induced by SPIO nanoparticles, the expression of LC3 and p62 proteins was evaluated by western blotting (Fig. 4A) and the levels were then quantified (Fig. 4B and C). After adding CLI095, a TRL4 signaling inhibitor, the expression of LC3-II and p62 could be inhibited in the SPIO nanoparticle treated macrophages at both mRNA and protein levels (Fig. 4D). It indicated that the macrophage autophagy induced by SPIO nanoparticles was triggered by the activation of Toll-like receptor (TLR)4 leading to an up-regulation of p62, which was different from the classic autophagy pathway that promotes autophagy by reducing the expression of p62 [24].

### Inflammatory responses related to SPIO-induced macrophage autophagy

**Figure 5.**
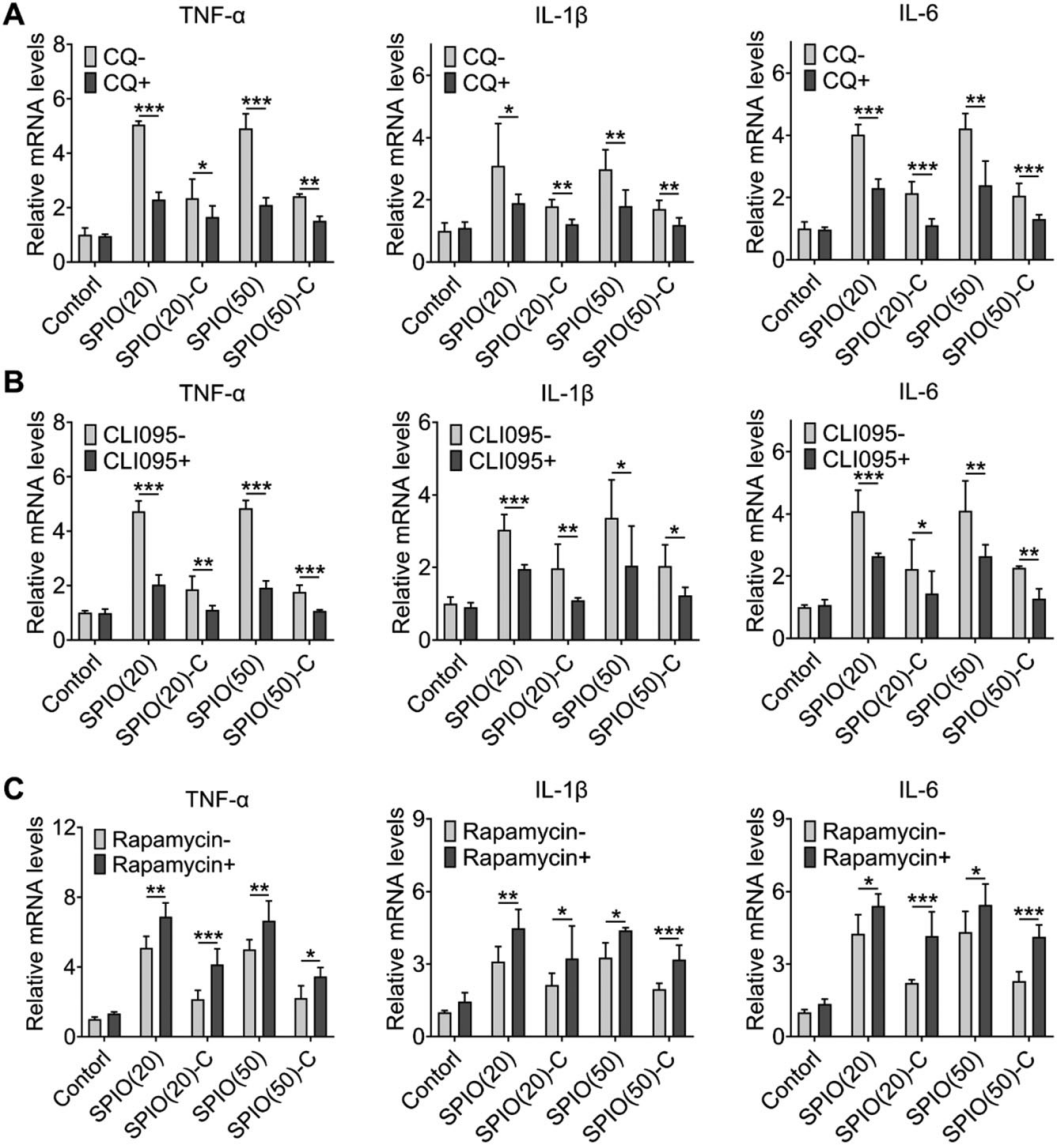
Investigation of the correlation between autophagy and inflammation. RT-qPCR test of TNF-α, IL-1β and IL-6 secreted by Raw264.7 pretreated with CQ (**A**), CLI-095 (**B**) and rapamycin (**C**) for 24 h. Values are expressed as the mean, *n* = 4. Bars show SD. *P < 0.05; **P < 0.01; ***P < 0.001

Recent studies have revealed that autophagy and inflammation are highly correlated and have mutual regulation for each other [25–27]. There are various inflammatory mediators that have effects on autophagy, including cytokines, reactive oxygen species and inflammatory transcription factors, and in the meanwhile, autophagy can regulate inflammatory responses through TLR and Nod-like receptor signaling pathways [3, 28]. Autophagy not only has influence on the pathological process of inflammatory diseases, but also can be finely tuned to achieve the treatment of inflammatory diseases [29]. SPIO-induced macrophage autophagy was proved to be activated through TLR4 signaling pathway, which has a relationship to the expression of inflammatory cytokines [24]. Therefore, we investigated the changes of inflammatory response with inhibition or promotion of macrophage autophagy through detecting the secretion of TNF-α, IL-1β and IL-6 of SPIO nanoparticles-treated Raw264.7 cells using reverse transcription and quantitative real-time polymerase chain reaction (RT-qPCR). Autophagy inhibitors (CQ and CLI-095) and an enhancer (rapamycin) were introduced to observe changes in the secretion of inflammatory cytokines. The expression levels of TNF-α, IL-1β and IL-6 of Raw264.7 cells pretreated with CQ and CLI-095 were reduced (Fig. 5A and B), and Raw264.7 cells pretreated with rapamycin produced significantly higher levels of inflammatory cytokines mentioned above (Fig. 5C). In general, compared with the control group treated by normal saline, the secretion of TNF-α in the group treated by bare SPIO nanoparticles increased five times, the secretion of IL-1β increased three times and the secretion of IL-6 increased four times. Although the secretion of the aforementioned inflammatory cytokines in the group treated by SPIO-C nanoparticles also increased, compared with the bare SPIO nanoparticles treatment group, TNF-α secretion was reduced by about 60%, IL-1β secretion was reduced by about 40% and IL-6 secretion was reduced by about 45%, which is consistent with the trend of autophagy induced by SPIO nanoparticles. Based on the above results, it can be suggested that the process of autophagy was positively correlated with the secretion of inflammatory cytokines. Although the relationship between autophagy and inflammatory response has been briefly studied, more specific signaling pathways are still unclear. Application of genomics and proteomics research strategies will be helpful to understand the mechanism of autophagy in inflammation and immune response, and it may bring new strategies to treat inflammatory diseases by the manipulation of autophagy.

### Evaluation of SPIO nanoparticle induced inflammation and autophagy *in vivo*

**Figure 6.**
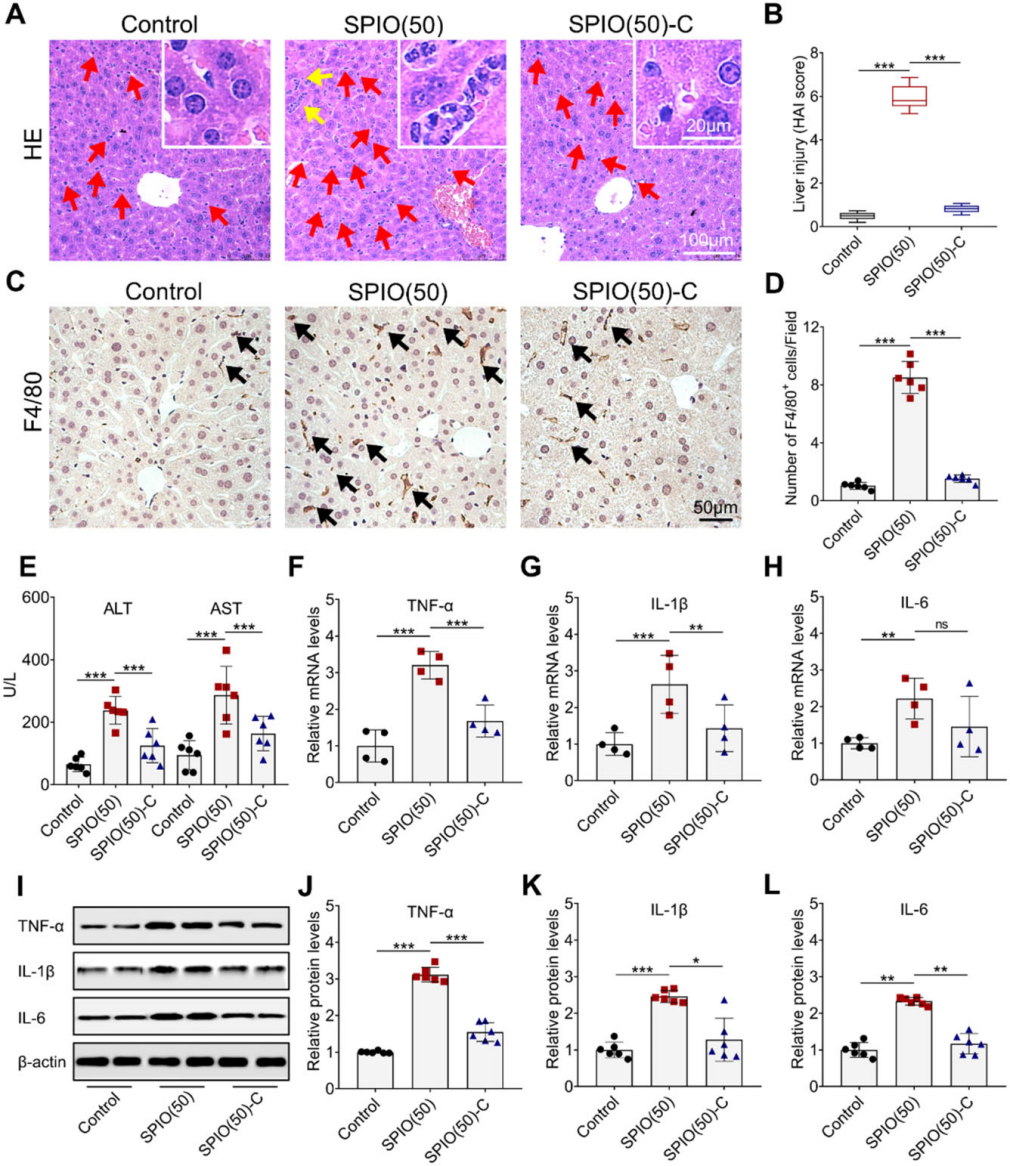
Effects of SP IO nanoparticles on the liver inflammation. (**A**) HE staining of liver section (Kupffer cells and the focal necrosis of hepatocytes are marked by arrows; the partial enlarged image is placed in the upper right corner). (**B**) Analysis of liver injury by HAI. (**C**) F4/80 staining of liver section (macrophages with high expression of F4/80 are marked by arrows). (**D**) Quantification of F4/80+ cells. (**E**) Evaluation of ALT and AST to determine the degree of liver injury. Expression of T NF-α (**F**), IL-1β (**G**) and IL-6 (**H**) in the liver detected by RT-qP CR. (**I**) Western blot analysis of T NF-α, IL-1β and IL-6 in the liver treated with SP IO nanoparticles for 24 h, β-actin was detected as a loading control. Densitometry analysis for quantifying the levels of T NF-α (**J**), IL-1β (**K**) and IL-6 (**L**). Values are expressed as the mean, n ≥ 4. Bars show SD. *P < 0.05; **P < 0.01; ***P < 0.001; ns, not significant

Nanoparticles with a particle size of 50 nm are more likely to be retained in the RES organs than those with a particle size of 20 nm [30], so SPIO(50) and SPIO(50)-C were intravenously injected into mice for evaluation of the systemic toxicity. Liver tissue was collected after 24 h of SPIO nanoparticle injection, and then embedded, sectioned and stained with hematoxylin and eosin (HE) for further studies (Fig. 6A). The increase in the number of Kupffer cells (marked by red arrows) and the appearance of focal necrosis of hepatocytes (marked by yellow arrows) were found in the group treated with SPIO(50), indicating a stronger inflammatory response in the liver. In comparison, abnormal increase of Kupffer cells and focal necrosis of hepatocytes could not be observed in the groups treated with SPIO(50)-C and saline. Thus, the inflammation induced by SPIO could be reduced by grafting carboxyl groups on the material surface. Histological activity index (HAI) score findings matched histological findings [31]. The HAI score of SPIO(50) was markedly higher than that of SPIO(50)-C (Fig. 6B). Then, the macrophages in the liver were stained by F4/80 (marked by black arrows) (Fig. 6C) [32], and the increase in the number of macrophages was observed when comparing to the groups treated with SPIO(50)-C and saline (Fig. 6D). Then, alanine aminotransferase (ALT) and aspartate aminotransferase (AST), biochemical parameters of liver function, were evaluated (Fig. 6E), indicating that the grafting of carboxyl groups may provide a protective effect on the liver [33]. Expression of TNF-α, IL-1β and IL-6, in the liver was further detected by RT-qPCR. The expression of inflammatory cytokines in the liver treated with the SPIO(50) sample was significantly higher than that treated with saline. After grafting carboxyl groups on SPIO nanoparticles, the secretion of TNF-α was reduced by 48% (Fig. 6F), the secretion of IL-1β was reduced by 46% (Fig. 6G), and the secretion of IL-6 was reduced by 34%, comparing to bare SPIO nanoparticles (Fig. 6H). The western blot analysis of the expression of above inflammatory cytokines in the liver was also conducted (Fig. 6I). Compared with the SPIO(50) treatment group, the expression of TNF-α in the group treated with SPIO(50)-C decreased by 50% (Fig. 6J), the expression of IL-1β decreased by 48% (Fig. 6K) and the expression of IL-6 decreased by 50% (Fig. 6L), which showed similar results with RT-qPCR.

**Figure 7.**
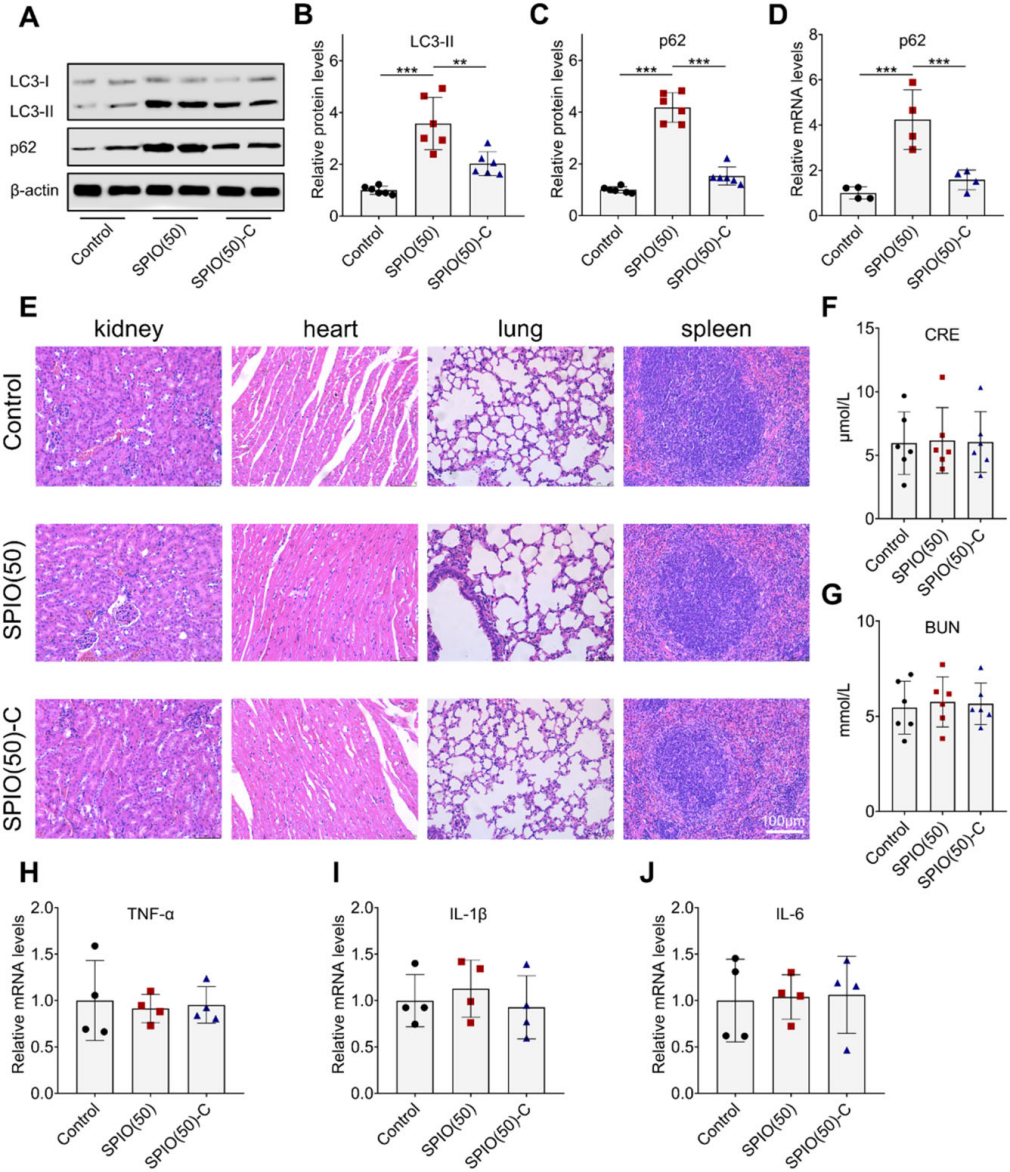
*In vivo* evaluation of SPIO nanoparticles. (**A**) Western blot analysis of LC3 and p62 in the liver treated with SPIO nanoparticles for 24 h, β-actin was detected as a loading control. Densitometry analysis for quantifying the levels of LC3-II (**B**) and p62 (**C**). (**D**) RT-qPCR analysis of p62 in the liver. (**E**) HE staining of the sections of kidney, heart, lung and spleen. Evaluation of CRE (**F**) and BUN (**G**) to determine the degree of kidney injury. RT-qPCR test of TNF-α (**H**), IL-1β (**I**) and IL-6 (**J**) in the kidney. Values are expressed as the mean, *n* ≥ 4. Bars show SD. **P < 0.01; ***P < 0.001

Additionally, evaluation of autophagy by western blot analysis of LC3-II and p62 in liver was performed (Fig. 7A). The expression of LC3-II and p62 in the SPIO(50)-C treated group was significantly decreased by 43% and 63% compared with that in the SPIO(50) treated group, indicating that the down-regulation of autophagy may be related to the down-regulation of inflammation (Fig. 7B and C). Then, the RT-qPCR confirmed the above results (Fig. 7D). Kidney, heart, lung and spleen tissues were also harvested and stained with HE. Both types of SPIO nanoparticles have no obvious impact on these organs (Fig. 7E). Besides, creatinine (CRE) and blood urea nitrogen (BUN), the indicators of renal function evaluation, were tested to determine whether the kidney was affected (Fig. 7F and G) [34]. The presentation of CRE and BUN in the mice treated with the two materials showed no significantly different and both are within the normal range. The RT-qPCR analysis was applied on the kidney, and there was no significant difference between the different groups, indicating the SPIO nanoparticles had no obvious cytotoxic effect on the kidneys at this dosage level (Fig. 7H–J).

**Figure 8.**
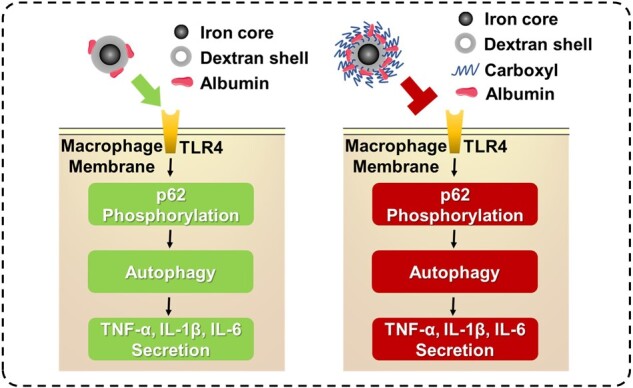
Inhibition of SPIO nanoparticles-induced autophagy and inflammatory cytokine secretion by grafting carboxyl groups

In this study, we grafted carboxyl groups on the surface of SPIO nanoparticles, and verified their improved biosafety from the perspective of macrophage autophagy. We observed that SPIO-C nanoparticles induced a lower degree of macrophage autophagy than unmodified SPIO nanoparticles. SPIO-induced macrophage autophagy triggered by activating TLR4 signaling pathway was shown to be positively related to inflammatory response, so carboxylation could be considered as a modification method to reduce the inflammation caused by SPIO nanoparticles (Fig. 8). However, the mechanism of the reduced macrophage autophagy achieved by carboxylation of SPIO nanoparticles remained to be elucidated. The adsorption of serum proteins on nanoparticles after injected into the body may be the key point to explain this phenomenon, and it was verified that more serum proteins could adsorb on SPIO-C nanoparticles. In short, the effect of carboxylation on reducing SPIO-induced macrophage autophagy and inflammation has been observed, which provide some insights for design of better nanomaterials for biomedical applications.

## Conclusion

In conclusion, a simple method to graft carboxyl groups on SPIO was proposed, and the difference between the bioeffect in macrophage induced by SPIO and SPIO-C nanoparticles was compared. Various experiments on Raw264.7 cells, including western blot, RT-qPCR and etc., had revealed that grafting carboxyl groups on the surface of SPIO could help reduce autophagy and inflammation, which was mostly like due to more adsorption of serum proteins on the carboxylated nanoparticle surface. Animal experiments have further verified that the increased biosafety of SPIO nanoparticles after grafting carboxyl groups. Due to the significant effect on inflammation, autophagy should undoubtedly be considered as an indispensable part of the biosafety evaluation of nanomaterials, and we used a simple method based on regulating macrophage autophagy to improve the biosafety of nanomaterials, which provides more options for the design of biomedical nanomaterials.

## Supplementary data

Supplementary data are available at *REGBIO* online.

## Funding 

This work was financially supported by the National Natural Science Foundation of China (NSFC, No. 51903174 and 52073192) and the Innovative Research Groups of the National Natural Science Foundation of China (81621003).

*Conflict of interest statement*. None declared.

## Supplementary Material

rbac018_Supplementary_Data
