# Supplementary material for: Surface carboxylation of iron oxide nanoparticles brings reduced macrophage inflammatory response through inhibiting macrophage autophagy
Source: Regen Biomater. 2022 Apr 20;9:rbac018. doi: 10.1093/rb/rbac018 (PMC9164630; doi:10.1093/rb/rbac018)
Supplement: rbac018_Supplementary_Data [file rbac018_supplementary_data.docx]

**Supplementary data**

**Carboxyl modification ameliorates superparamagnetic iron oxide nanoparticles-induced liver inflammatory response through inhibiting macrophage autophagy**

Di Deng^1^, Shengxiang Fu^1^, Zhongyuan Cai^1^, Xiaomin Fu^1^, Rongrong Jin^1*^, Hua Ai^1, 2*^


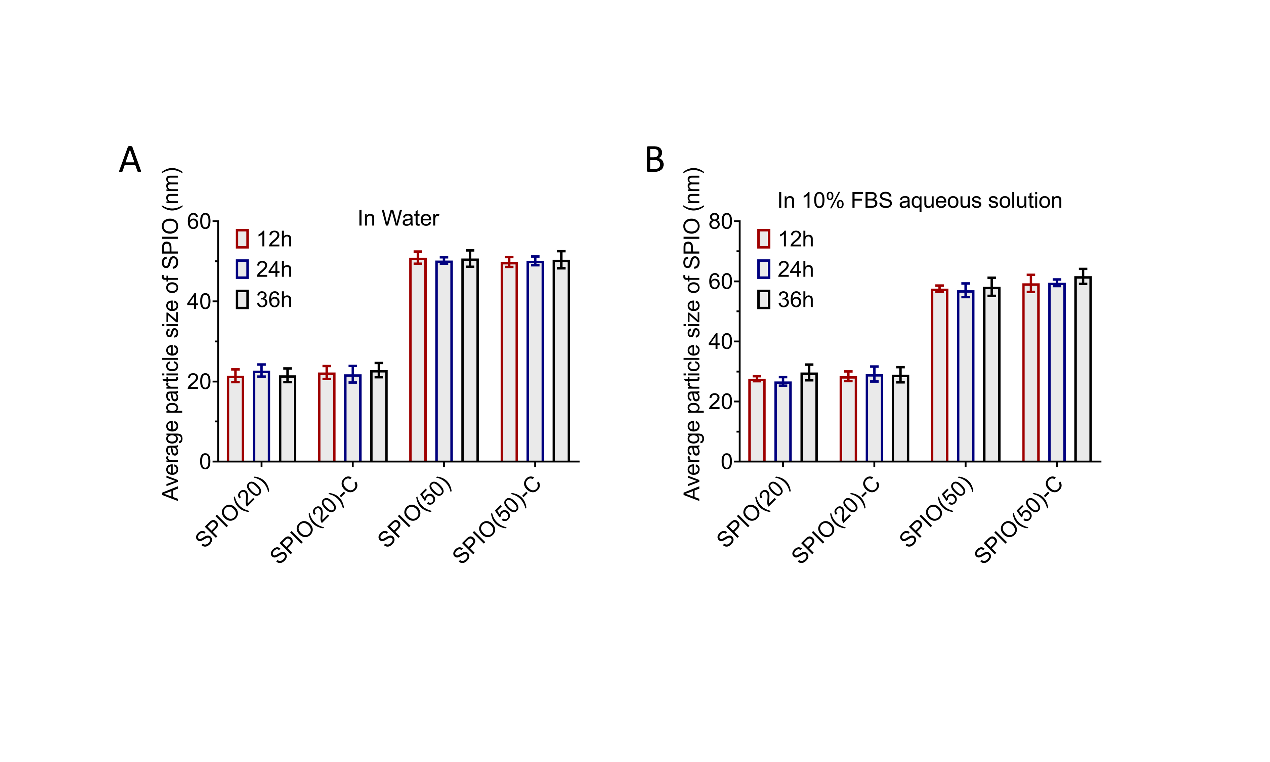


Figure S1. Particle size of synthesized SPIO nanoparticles in water (A) and in 10% FBS aqueous solution (B) within 36 h.

| **Supplementary Table 1.** Identification of adsorbed serum proteins on SPIO(50) surface | | | | | | | | |
| --- | --- | --- | --- | --- | --- | --- | --- | --- |
| Gene | Sum PEP Score | Coverage | # Peptides | # PSMs | # AAs | MW [kDa] | calc. pI | emPAI |
| MYH9 | 42.79895012 | 16.33587786 | 26 | 33 | 1965 | 227.063 | 5.59 | 0.648 |
| A2M | 38.48912488 | 17.68211921 | 22 | 25 | 1510 | 167.47 | 6.02 | 0.756 |
| ALB | 30.01420709 | 27.18286656 | 18 | 26 | 607 | 69.278 | 6.18 | 1.537 |
| FN1 | 25.35786873 | 6.612841215 | 11 | 14 | 2601 | 285.068 | 5.9 | 0.3 |
| KRT14 | 24.81024229 | 24.46601942 | 13 | 14 | 515 | 55.861 | 5.27 | 1.448 |
| C4A | 24.39298089 | 10.45376221 | 14 | 19 | 1741 | 192.676 | 7.43 | 0.398 |
| HBA | 23.67906807 | 57.04225352 | 6 | 13 | 142 | 15.175 | 8.44 | 11.915 |
| LOC107131209 | 19.39403358 | 9.048991354 | 12 | 18 | 1735 | 192.1 | 7.52 | 0.385 |
| KRT5 | 19.23535157 | 15.9733777 | 12 | 14 | 601 | 62.898 | 7.81 | 1.202 |
| APOE | 19.00346607 | 33.13069909 | 8 | 16 | 329 | 37.4 | 6.3 | 1.848 |
| KRT10 | 16.8913742 | 13.30798479 | 9 | 15 | 526 | 54.816 | 5.07 | 1.346 |
| C3 | 16.64688879 | 7.525586996 | 11 | 13 | 1661 | 187.135 | 6.84 | 0.286 |
| KRT3 | 16.31293065 | 8.121019108 | 7 | 12 | 628 | 64.129 | 8.38 | 0.778 |
| KRT1 | 3.575244605 | 4.290429043 | 2 | 2 | 606 | 63.127 | 8.46 | 0.133 |
| KRT17 | 12.58598569 | 13.15192744 | 7 | 8 | 441 | 48.712 | 5.15 | 0.719 |
| KRT6A | 11.94537724 | 10.33274956 | 7 | 9 | 571 | 60.767 | 8.09 | 0.585 |
| LOC107131172 | 11.93119496 | 43.44827586 | 6 | 9 | 145 | 15.796 | 6.68 | 2.831 |
| SERPINC1 | 10.21146104 | 11.50943396 | 5 | 9 | 530 | 60.031 | 8.68 | 0.468 |
| KRT2 | 9.588800913 | 5.331179321 | 4 | 8 | 619 | 64.399 | 8.56 | 0.307 |
| THBS1 | 9.548959697 | 5.446808511 | 6 | 8 | 1175 | 129.795 | 4.93 | 0.199 |
| F13A1 | 9.132368338 | 9.153005464 | 5 | 5 | 732 | 82.752 | 6.06 | 0.334 |
| KRT79 | 8.006060344 | 6.168224299 | 4 | 6 | 535 | 57.685 | 7.46 | 0.334 |
| GN=ITIH2 | 7.764679809 | 9.196617336 | 6 | 6 | 946 | 106.12 | 7.94 | 0.264 |
| SERPIND1 | 7.322217778 | 8.303886926 | 4 | 4 | 566 | 62.665 | 6.68 | 0.334 |
| APOA1 | 6.824747845 | 17.35849057 | 4 | 4 | 265 | 30.258 | 5.97 | 0.585 |
| TF | 6.705206302 | 8.948863636 | 5 | 5 | 704 | 77.688 | 7.17 | 0.265 |
| AHSG | 6.465820579 | 6.963788301 | 2 | 6 | 359 | 38.394 | 5.5 | 0.468 |
| PLG | 6.001371536 | 3.325123153 | 4 | 4 | 812 | 91.184 | 7.64 | 0.186 |
| SERPINA1 | 6.000324627 | 12.5 | 5 | 6 | 416 | 46.075 | 6.52 | 0.532 |
| TLN1 | 5.868717591 | 2.235294118 | 4 | 4 | 2550 | 270.58 | 6.09 | 0.06 |
| ITIH4 | 5.739045137 | 5.895196507 | 5 | 5 | 916 | 101.449 | 6.68 | 0.271 |
| FGG | 5.718395921 | 12.64108352 | 4 | 5 | 443 | 50.2 | 5.72 | 0.389 |
| ACTB | 5.22640886 | 14.4 | 4 | 5 | 375 | 41.71 | 5.48 | 0.492 |
| COL1A1 | 4.558640492 | 1.503759398 | 2 | 2 | 1463 | 138.854 | 5.78 | 0.053 |
| GSN | 4.543052968 | 3.546099291 | 3 | 4 | 846 | 92.027 | 6.86 | 0.239 |
| VTN | 4.282909388 | 11.97478992 | 4 | 4 | 476 | 53.541 | 6.28 | 0.52 |
| FGA | 4.250032603 | 2.549246813 | 2 | 3 | 863 | 94.675 | 6 | 0.134 |
| F2 | 2.747540778 | 4 | 2 | 2 | 625 | 70.53 | 6.44 | 0.129 |
| C9 | 2.448139812 | 3.649635036 | 2 | 2 | 548 | 61.958 | 5.9 | 0.145 |
| KRT24 | 2.370170582 | 4.952380952 | 2 | 2 | 525 | 55.095 | 5 | 0.155 |
| SERPINF2 | 2.2888939 | 3.861788618 | 2 | 2 | 492 | 54.676 | 5.72 | 0.212 |
| SERPINA5 | 2.254049801 | 9.158415842 | 2 | 2 | 404 | 45.268 | 9.36 | 0.222 |
| AFP | 2.247588895 | 2.786885246 | 2 | 2 | 610 | 68.543 | 6.29 | 0.122 |
| F5 | 2.056991543 | 0.949796472 | 2 | 2 | 2211 | 248.828 | 5.83 | 0.048 |
| ITIH3 | 1.780415474 | 1.234567901 | 1 | 1 | 891 | 99.489 | 5.9 | 0.05 |
| C7 | 1.528414946 | 1.18623962 | 1 | 1 | 843 | 92.929 | 7.2 | 0.042 |
| F13B | 1.457423524 | 1.664145234 | 1 | 1 | 661 | 75.118 | 6.76 | 0.062 |
| ENAH | 1.311936303 | 1.003764115 | 1 | 1 | 797 | 86.048 | 7.91 | 0.083 |
| CPS1 | 1.206698646 | 0.666666667 | 1 | 1 | 1500 | 164.636 | 6.73 | 0.027 |
| EEF1A2 | 1.175483672 | 2.375809935 | 1 | 1 | 463 | 50.438 | 9.03 | 0.096 |
| APOB | 1.143694134 | 0.284650755 | 1 | 1 | 4567 | 515.195 | 6.68 | 0.008 |
| CFB | 1.111259039 | 1.445466491 | 1 | 1 | 761 | 85.312 | 7.68 | 0.056 |
| KNG1 | 1.061780582 | 1.449275362 | 1 | 1 | 621 | 68.922 | 6.67 | 0.058 |
| CFH | 1.061480275 | 0.64516129 | 1 | 1 | 1240 | 140.877 | 6.8 | 0.031 |
| DSP | 1.056752875 | 0.31152648 | 1 | 1 | 2889 | 332.202 | 6.84 | 0.011 |
| MINDY4 | 1.042344833 | 1.441677588 | 1 | 1 | 763 | 84.394 | 6.96 | 0.054 |
| C4BPA | 1.020679303 | 1.31147541 | 1 | 1 | 610 | 68.841 | 6.38 | 0.062 |
| ALPK1 | 0.960982678 | 0.563607085 | 1 | 1 | 1242 | 138.381 | 6.33 | 0.03 |
| TUBA1D | 0.940058112 | 3.318584071 | 1 | 1 | 452 | 50.251 | 5.03 | 0.101 |
| ARHGEF11 | 0.933674075 | 0.686641698 | 1 | 1 | 1602 | 176.333 | 5.72 | 0.028 |
| C5 | 0.89245087 | 0.655933214 | 1 | 1 | 1677 | 188.677 | 6.61 | 0.022 |
| ECM1 | 0.87484417 | 1.757469244 | 1 | 1 | 569 | 63.137 | 6.99 | 0.072 |
| GC | 0.868702203 | 2.315789474 | 1 | 1 | 475 | 53.627 | 5.88 | 0.077 |

| **Supplementary Table 2.** Identification of adsorbed serum proteins on SPIO(50)-C surface | | | | | | | | |
| --- | --- | --- | --- | --- | --- | --- | --- | --- |
| Gene | Sum PEP Score | Coverage | # Peptides | # PSMs | # AAs | MW [kDa] | calc. pI | emPAI |
| C3 | 24.84531641 | 15.11137869 | 16 | 16 | 1661 | 187.135 | 6.84 | 0.398 |
| MYH9 | 65.51245996 | 22.08651399 | 30 | 34 | 1965 | 227.063 | 5.59 | 0.77 |
| FN1 | 65.2577793 | 17.72395233 | 26 | 31 | 2601 | 285.068 | 5.9 | 0.833 |
| C5 | 42.76327252 | 18.72391175 | 22 | 27 | 1677 | 188.677 | 6.61 | 0.769 |
| APOB | 35.65220374 | 5.977665864 | 20 | 21 | 4567 | 515.195 | 6.68 | 0.18 |
| LOC107131209 | 34.94902945 | 14.81268012 | 18 | 24 | 1735 | 192.1 | 7.52 | 0.668 |
| A2M | 33.91816321 | 18.74172185 | 18 | 22 | 1510 | 167.47 | 6.02 | 0.668 |
| ITIH2 | 23.6977848 | 12.68498943 | 7 | 11 | 946 | 106.12 | 7.94 | 0.536 |
| ALB | 22.52432155 | 23.723229 | 13 | 20 | 607 | 69.278 | 6.18 | 1.085 |
| HBA | 17.66474346 | 43.66197183 | 4 | 9 | 142 | 15.175 | 8.44 | 6.743 |
| C9 | 12.83006847 | 18.43065693 | 8 | 9 | 548 | 61.958 | 5.9 | 0.84 |
| SERPINC1 | 12.02555553 | 17.16981132 | 7 | 9 | 530 | 60.031 | 8.68 | 0.778 |
| VTN | 10.24609975 | 14.07563025 | 5 | 6 | 476 | 53.541 | 6.28 | 0.688 |
| TLN1 | 9.633879794 | 3.137254902 | 5 | 5 | 2550 | 270.58 | 6.09 | 0.076 |
| SERPING1 | 9.367757421 | 13.24786325 | 4 | 5 | 468 | 51.74 | 6.76 | 0.73 |
| GSN | 9.450215866 | 8.274231678 | 5 | 6 | 846 | 92.027 | 6.86 | 0.379 |
| FGG | 9.232225884 | 20.99322799 | 6 | 7 | 443 | 50.2 | 5.72 | 0.778 |
| SERPINA5 | 8.791366271 | 14.6039604 | 3 | 4 | 404 | 45.268 | 9.36 | 0.35 |
| SERPINA1 | 5.180508661 | 13.94230769 | 4 | 4 | 416 | 46.075 | 6.52 | 0.407 |
| AHSG | 5.341709045 | 17.54874652 | 4 | 7 | 359 | 38.394 | 5.5 | 0.668 |
| TF | 7.655412621 | 8.806818182 | 4 | 4 | 704 | 77.688 | 7.17 | 0.207 |
| F13A1 | 7.350569835 | 9.426229508 | 5 | 5 | 732 | 82.752 | 6.06 | 0.334 |
| GFM2 | 8.282835953 | 2.316602317 | 1 | 6 | 777 | 85.977 | 7.14 | 0.166 |
| F2 | 3.326462107 | 5.6 | 2 | 3 | 625 | 70.53 | 6.44 | 0.199 |
| CFB | 5.862123649 | 9.592641261 | 4 | 6 | 761 | 85.312 | 7.68 | 0.245 |
| ITIH1 | 5.828112703 | 5.629139073 | 3 | 3 | 906 | 101.173 | 7.4 | 0.162 |
| SERPIND1 | 5.768697389 | 8.657243816 | 4 | 4 | 566 | 62.665 | 6.68 | 0.334 |
| ACTB | 5.522964677 | 19.73333333 | 4 | 4 | 375 | 41.71 | 5.48 | 0.492 |
| THBS4 | 5.075111789 | 5.827263267 | 4 | 4 | 961 | 105.879 | 4.68 | 0.233 |
| COL1A1 | 2.410944469 | 1.503759398 | 1 | 1 | 1463 | 138.854 | 5.78 | 0.026 |
| C7 | 4.128559447 | 4.151838671 | 3 | 3 | 843 | 92.929 | 7.2 | 0.131 |
| C6 | 3.760965003 | 3.315508021 | 2 | 3 | 935 | 105.047 | 6.87 | 0.136 |
| ATP5F1A | 3.140607087 | 2.350813743 | 1 | 3 | 553 | 59.653 | 9.19 | 0.133 |
| PLG | 3.755919491 | 4.187192118 | 3 | 3 | 812 | 91.184 | 7.64 | 0.136 |
| KRT7 | 3.538385787 | 4.935622318 | 2 | 2 | 466 | 51.546 | 5.97 | 0.141 |
| TUBB1 | 3.532815475 | 4.99001996 | 2 | 2 | 501 | 55.347 | 5.62 | 0.179 |
| APOA1 | 3.769621082 | 10.18867925 | 2 | 2 | 265 | 30.258 | 5.97 | 0.259 |
| KRT2 | 3.112992548 | 4.846526656 | 3 | 3 | 619 | 64.399 | 8.56 | 0.113 |
| C8A | 3.083019953 | 3.395585739 | 1 | 1 | 589 | 66.234 | 6.64 | 0.068 |
| CFH | 3.023372702 | 1.370967742 | 1 | 2 | 1240 | 140.877 | 6.8 | 0.063 |
| ACTC1 | 2.983755561 | 10.71428571 | 3 | 3 | 392 | 43.541 | 5.44 | 0.413 |
| KRT1 | 2.964264936 | 4.95049505 | 3 | 3 | 606 | 63.127 | 8.46 | 0.133 |
| C8B | 2.884336777 | 5.423728814 | 2 | 2 | 590 | 66.642 | 8.03 | 0.155 |
| AHCY | 2.665130852 | 4.861111111 | 2 | 2 | 432 | 47.607 | 6.29 | 0.202 |
| C8G | 2.514278432 | 7.484407484 | 2 | 2 | 481 | 50.802 | 10.87 | 0.179 |
| POSTN | 2.786482243 | 1.674641148 | 1 | 1 | 836 | 93.135 | 7.37 | 0.056 |
| SERPINF2 | 2.362196571 | 4.674796748 | 2 | 2 | 492 | 54.676 | 5.72 | 0.212 |
| C1QB | 2.276872841 | 5.668016194 | 1 | 1 | 247 | 26.383 | 9.48 | 0.155 |
| PGLYRP1 | 2.235599677 | 7.368421053 | 1 | 1 | 190 | 21.05 | 9.5 | 0.233 |
| COMP | 2.224608028 | 1.851851852 | 1 | 1 | 756 | 82.31 | 4.61 | 0.066 |
| THBS1 | 2.176143682 | 1.787234043 | 2 | 2 | 1175 | 129.795 | 4.93 | 0.062 |
| KRT79 | 2.046641262 | 3.925233645 | 2 | 2 | 535 | 57.685 | 7.46 | 0.155 |
| KRT10 | 2.016494367 | 3.802281369 | 2 | 2 | 526 | 54.816 | 5.07 | 0.186 |
| FGA | 1.956301421 | 1.042873696 | 1 | 2 | 863 | 94.675 | 6 | 0.087 |
| ECM1 | 1.807845295 | 4.569420035 | 2 | 2 | 569 | 63.137 | 6.99 | 0.15 |
| APOE | 1.793122335 | 7.294832827 | 2 | 2 | 329 | 37.4 | 6.3 | 0.233 |
| ITIH3 | 1.758514534 | 2.132435466 | 2 | 2 | 891 | 99.489 | 5.9 | 0.103 |
| FBLN1 | 1.257982253 | 1.699716714 | 1 | 1 | 706 | 77.434 | 5.06 | 0.064 |
| CDK5RAP1 | 0.902048929 | 2.177554439 | 1 | 1 | 597 | 67.193 | 8.13 | 0.066 |
| ADIPOQ | 1.118729496 | 4.573170732 | 1 | 1 | 328 | 35.072 | 5.6 | 0.136 |
| GAPDH | 0.910448117 | 3.989361702 | 1 | 1 | 376 | 40.494 | 9.13 | 0.101 |
| FLNA | 0.897223385 | 0.41556479 | 1 | 1 | 2647 | 280.752 | 6.11 | 0.015 |
| NDUFV1 | 0.881735274 | 1.782531194 | 1 | 1 | 561 | 60.322 | 8.87 | 0.075 |
| TTC21B | 0.81417464 | 1.747720365 | 1 | 1 | 1316 | 151.057 | 6.95 | 0.028 |
| KRT14 | 0.809388202 | 1.747572816 | 1 | 1 | 515 | 55.861 | 5.27 | 0.066 |
| COL6A3 | 0.787812396 | 0.287356322 | 1 | 1 | 3132 | 339.377 | 6.37 | 0.012 |
| CPS1 | 0.751781439 | 0.666666667 | 1 | 1 | 1500 | 164.636 | 6.73 | 0.027 |
| ENAH | 0.748848657 | 1.003764115 | 1 | 2 | 797 | 86.048 | 7.91 | 0.083 |
| HEATR5B | 0.693146251 | 0.966183575 | 1 | 1 | 2070 | 224.145 | 7.09 | 0.02 |
| AFP | 0.597910649 | 1.475409836 | 1 | 1 | 610 | 68.543 | 6.29 | 0.059 |
